# Those Chairs Again

**Published:** 1897-09

**Authors:** 


THOSE CHAIRS AGAIN.—The Texas Association of Railway Surgeons in session recently in Galveston, spoke out in advocacy of the claims of Texas physicians to the vacant professorships in the Texas Medical College. The Southwestern Texas Medical Association, at Houston, also puts itself on record as emphatic in the belief that Texas physicians should be teachers in the Texas Medical College, especially as Texas tax-payers "set up" the medical education for the Texas boys. We do not hesitate to say that if a canvass of the Texas profession could be made it would be found that they are unanimously of the same belief. As our neighbor, the *Texas Medical News*, very truly says, "the medical profession of Texas can make or mar the school." Its tenure is not yet so secure that Texas physicians who, having in the beginning been invited to accept positions, and who have done their best, can be virtually kicked out, their chairs declared vacant without notice—no charges preferred, no cause assigned—to make room perhaps for aliens, without some sign of protest. They will hardly consent to take back seats in favor of young graduates of Johns-Hopkins University or of Jefferson or even of Europe, and still extend to the school their cordial good will and patronage. As a matter of policy, as well as of right and justice, the teachers for the school should be taken from the ranks of the home profession. It would be the merest subterfuge to try to justify any other course by saying that the college needs and must have up to date men. The best physicians of Texas are up to date men. The record of their work will compare favorably with that of the best physicians of Europe or elsewhere. We believe the regents will make a serious, nay, a fatal mistake if they select any more teachers from abroad.

---

"OUR hot and cold contemporary of the Red Back."—*S. W. Medical Record*, Houston. Thanks, Brother Red. How is our luke warm little friend, the *Record*, by this time?

